# Supplementary material for: A Critical View of the Application of the APEX Software (Aqueous Photochemistry of Environmentally-Occurring Xenobiotics) to Predict Photoreaction Kinetics in Surface Freshwaters
Source: Molecules. 2019 Dec 18;25(1):9. doi: 10.3390/molecules25010009 (PMC7017383; doi:10.3390/molecules25010009)
Supplement: Supplementary file 1 [file molecules-25-00009-s001.zip › 3.2.4_gcc-4.4.0.zip!/3.2.4_gcc-4.4.0/3.2.4_gcc-4.4.0/doc/octave/html/interpreter/Style-Tips.html]

Style Tips - Untitled


Next: Coding Tips,
Up: Tips and Standards


---

### C.1 Writing Clean Octave Programs

Here are some tips for avoiding common errors in writing Octave code
intended for widespread use:

- Since all global variables share the same name space, and all functions
  share another name space, you should choose a short word to distinguish
  your program from other Octave programs. Then take care to begin the
  names of all global variables, constants, and functions with the chosen
  prefix. This helps avoid name conflicts.

  If you write a function that you think ought to be added to Octave under
  a certain name, such as `fiddle_matrix`, don't call it by that name
  in your program. Call it `mylib_fiddle_matrix` in your program,
  and send mail to maintainers@octave.org suggesting that it
  be added to Octave. If and when it is, the name can be changed easily
  enough.

  If one prefix is insufficient, your package may use two or three
  alternative common prefixes, so long as they make sense.

  Separate the prefix from the rest of the symbol name with an underscore
  \_. This will be consistent with Octave itself and with most
  Octave programs.- When you encounter an error condition, call the function `error`
    (or `usage`). The `error` and `usage` functions do not
    return.
    See Errors.- Please put a copyright notice on the file if you give copies to anyone.
      Use the same lines that appear at the top of the function files
      distributed with Octave. If you have not signed papers to assign the
      copyright to anyone else, then place your name in the copyright notice.
